# Genomic analyses of African *Trypanozoon* strains to assess evolutionary relationships and identify markers for strain identification

**DOI:** 10.1371/journal.pntd.0005949

**Published:** 2017-09-29

**Authors:** Joshua Brian Richardson, Kuang-Yao Lee, Paul Mireji, John Enyaru, Mark Sistrom, Serap Aksoy, Hongyu Zhao, Adalgisa Caccone

**Affiliations:** 1 Department of Ecology and Evolutionary Biology, Yale University, New Haven, CT, United States of America; 2 Yale School of Public Health, Yale University, New Haven, CT, United States of America; 3 Biotechnology Research Institute, Kenya Agricultural and Livestock Research Organization, Kikuyu, Kenya; 4 School of Biological Sciences, Makerere University, Kampala, Uganda; 5 School of Natural Sciences, UC Merced, Merced, CA, United States of America; Institut de recherche pour le developpement, FRANCE

## Abstract

African trypanosomes of the sub-genus *Trypanozoon)* are eukaryotic parasitesthat cause disease in either humans or livestock. The development of genomic resources can be of great use to those interested in studying and controlling the spread of these trypanosomes. Here we present a large comparative analysis of *Trypanozoon* whole genomes, 83 in total, including human and animal infective African trypanosomes: 21 *T*. *brucei brucei*, 22 *T*. *b*. *gambiense*, 35 *T*. *b*. *rhodesiense* and 4 *T*. *evansi* strains, of which 21 were from Uganda. We constructed a maximum likelihood phylogeny based on 162,210 single nucleotide polymorphisms (SNPs.) The three *Trypanosoma brucei* sub-species and *Trypanosoma evansi* are not monophyletic, confirming earlier studies that indicated high similarity among *Trypanosoma* “sub-species”. We also used discriminant analysis of principal components (DAPC) on the same set of SNPs, identifying seven genetic clusters. These clusters do not correspond well with existing taxonomic classifications, in agreement with the phylogenetic analysis. Geographic origin is reflected in both the phylogeny and clustering analysis. Finally, we used sparse linear discriminant analysis to rank SNPs by their informativeness in differentiating the strains in our data set. As few as 84 SNPs can completely distinguish the strains used in our study, and discriminant analysis was still able to detect genetic structure using as few as 10 SNPs. Our results reinforce earlier results of high genetic similarity between the African *Trypanozoon*. Despite this, a small subset of SNPs can be used to identify genetic markers that can be used for strain identification or other epidemiological investigations.

## Author summary

Trypanosomes are a major health threat to the people and livestock of Sub-Saharan Africa. Building genomic resources and understanding the genetic structure of these parasites will aid researchers trying to control their spread. To this end, we compared the genomes from 83 trypanosome strains, identifying 162,210 single nucleotide polymorphisms (SNPs) between them. Our analysis shows high genetic similarity between the trypanosomes, and confirms earlier results indicating that the traditional taxonomic classifications do not correspond well with genetic data. Further, we demonstrate that, despite the high genetic similarity, each strain in the study can be distinguished using as few as 84 SNPs, suggesting that a small number of SNPs can be useful for tracking and classifying populations of African trypanosomes.

## Introduction

Trypanosomes are single-celled, eukaryotic parasites of mammalian bloodstreams that are a major health and economic burden on communities with endemic circulating strains. This is especially true in sub-Saharan Africa, where trypanosomes belonging to the species *Trypanosoma brucei* are vectored between human and animal hosts by the tsetse fly. *Trypanosoma brucei brucei* (*Tbb*) causes nagana in livestock, while *Trypanosoma brucei gambiense* (*Tbg*) causes chronic sleeping sickness and *Trypanosoma brucei rhodesiense* (*Tbr*) causes acute sleeping sickness in humans. This species, along with *Trypanosoma evansi* (*Tev*) and *Trypanosoma equiperdum* (*Teq*) comprise the sub-genus *Trypanozoon*. Both *Tev* and *Teq* are also found outside of sub-Saharan Africa and are recognized as being highly similar to *Trypanosoma brucei* [1,2]. *Tev* causes surra, a disease of livestock, and *Teq* causes dourine, which affects horses. Most of the African *Trypanozoon* taxa are morphologically indistinguishable, with the main differences between them being the host in which the trypanosome causes disease or the insect vector that enables their distribution (with the exception of *Teq*, which has lost its dependence on an arthropod host). In addition, both *Tev* and *Teq* have lost all or part of their kinetoplastid DNA (analogous to mitochondrial DNA in other eukaryotes [2]). Both *Tbr* and *Tbg* have evolved distinct mechanisms of evading trypanolytic factors in human blood [3,4,5]. Despite some similarities, and the ability for some groups to recombine [6], each group has developed divergent phenotypes that affect their range and impact on humans and livestock.

The African trypanosomes belonging to the *Trypanozoon* sub-genus were first isolated in the late 19th and early 20th centuries [6]. Being morphologically similar, and without the aid of molecular techniques, early taxonomic classifications were therefore based on host range and symptoms caused by infection. *Tbb* is found throughout sub-Saharan Africa, matching the range of the tsetse fly vector. *Tbr* is found in the Eastern portion of this range, and *Tbg* found in the West. *Teq* and *Tev*, however, have spread to Northern Africa, Asia and beyond. While a classification system based on infectious symptoms and geography is useful in a clinical setting, it does not necessarily reflect the actual phylogenetic relationship among trypanosomes in this group. Initially, it was not clear if the genetic differences between the groups represented deep phylogenetic divergence supporting a separate species classification, or were more representative of an alternative phenotype or sub-species. With the development and application of molecular tools, it has become increasingly clear that none of the *T*. *brucei* sub-species merits a separate species rank based on their genetic relatedness. Whole genome analyses of selected *Tev* and *Teq* strains showed the two groups are highly similar to *Tbb*, despite their dramatic range expansion and loss of functional kinetoplastids [7]. Similarly, molecular evidence suggests that *Tbr* is essentially a *Tbb* strain that has acquired the serum resistance-associated (SRA) gene. This transition apparently evolved multiple times from different *Tbb* strains [8,9,10,11]. Isolates of *Trypanosoma brucei gambiense* have been divided into *Tbg* group 1 (*Tbg-1*) and *Tbg* group 2 (*Tbg-2*). The first one comprises most *Tbg* isolates. They are genetically distinct from the single *Tbg-2* isolate found originally in Ivory Coast and now thought to be extinct [12,13]. Both *Tbg* types are more distinct from *Tbb* than *Tbr* still harbors a large amount of genetic similarities to it [8,11,14,15]. Isolates form *T*. *evansi* also harbor further genetic subdivisions, with *Tev* strains being classified as Type A or Type B, according to their mini-circle DNA sequences [2] and the presence of the RoTat1.2 gene [16]. Taken together, this evidence supports the idea that mutations altering host range and specificity have occurred relatively recently and are not uncommon. This highlights the need to monitor trypanosome populations of both human and non-human parasites, as these types of mutations in particular could have serious health and/or economic consequences. This is especially concerning in Uganda, which contains both the Eastern edge of the *Tbg-1* range and the Western edge of the *Tbr* range [17]. Because both prognosis and treatment of the two types of sleeping sickness caused by the two variants is different, accurate diagnosis is critical. The two ranges are less than 100km apart [17], and *Tbb* and the tsetse fly vector occur throughout the country, overlapping with the *Tbr* and *Tbg-1* ranges. Sleeping sickness cases caused by a co-infection of both *Tbg-1* and *Tbr* could be a possibility. The epidemiological consequences of the overlap in ranges are difficult to predict but would certainly pose challenges to healthcare professionals.

The purpose of the present study is twofold. First, we provide a large comparative genomic analysis of African trypanosomes of the sub-genus *Trypanozoon* by analyzing whole genome data from 83 strains from across sub-Saharan Africa, representing a range of human and animal infective types (S1 Table), and comprising 21 *Tbb*, 21 *Tbg-1*, 1 *Tbg-2*, 35 *Tbr* and 4 *Tev* strains (4 Type A and 1 type B). Nineteen of the *Tbg-1* strains were isolated from a hospital in the Democratic Republic of the Congo in response to a sleeping sickness outbreak [18]. Eleven of the newly sequenced strains, and 24 in total, are from Uganda, the only country where both *Tbr* and *Tbg-1* co-occur and where methods of strain discovery can be extremely useful [17]. This data set complements previous work that has sought to clarify the genetic relationships between the African trypanosomes [7,8,10,11, 19,20].

For the second aim, we used the genome data from these 83 strains in combination with a powerful statistical technique, sparse linear discriminant analysis (SLDA), to select a subset of SNPs to facilitate strain identification. SLDA calculates linkage disequilibrium across all SNPs and, unlike existing procedures which generally consider the association between a phenotype and a single SNP, simultaneously selects informative variants across the whole genome [21–25]. In this study, we used SLDA to evaluate the possibility of using a small subset of SNPs to classify *Trypanozoon* strains into genetic clusters, identified using whole genome data. Minimizing the number of SNPs necessary to diagnose specific strains of *T*. *brucei* has practical implications, as being able to define strains using a subset of markers significantly reduces the cost and effort to do so, allowing for the development of field portable diagnostics [26]. Selecting only the most informative SNPs through SLDA will still yield information reflective of the whole genome data and should prove useful from an epidemiological perspective.

## Methods

### Sequencing and SNP discovery

S1 Table lists the source and sub-species of all 83 trypanosome strains analyzed in this paper, along with the reference where their genome sequence was presented, if applicable. For all previously undescribed strains (except for STIB348TBABB, a derivative of Stib348), a diagnostic ITS1 PCR [27] was conducted to screen for *T*. *brucei* versus other African trypanosomes. A second PCR test for the presence of the serum resistance-associated (SRA) gene diagnostic of *Tbr* (following Radwanska et al. [28]) was also performed. A third PCR for the *Tbg* specific glycoprotein TgsGP [28] was conducted on SRA negative strains to distinguish *Tbg* from *Tbb*. The newly screened strains included 503_s, Cow248, Apendum, Dog157, Keko, LWO07A, LWO11A, LWO150A, LWO24A, and LWO30A. Whole genome sequencing and extraction was performed as described in [10], with all sequencing performed at the Yale Center for Genomic Analysis using the Illumina HiSeq 2000 platform. Raw sequences were quality checked with Fastqc [29] and aligned to the large chromosomes of the reference *Trypanosoma brucei brucei* strain 927 [30] genome, using Bowtie2 [31]. To prepare the data for SNP calling, duplicate read removal was performed using picard tool's MarkDuplicates tool. In addition, realignment around indels (insertions and deletions) was done using the Genome Analysis Toolkit's (GATK) IndelRealigner tool [32]. SNPs were called using GATK's HaplotypeCaller tool [33]. SNPs with a minor allele frequency of less than 0.05 and/or any missing data were filtered out using vcftools [34]. SNPs occurring in repetitive regions, as determined by RepeatMasker [35], or in the coding sequencing of variant surface glycoprotein genes (VSG), were excluded using R [36], as described in [36], due to the difficulty in accurately assigning SNPs to these regions. After all filtering steps, 162,210 SNPs were retained.

### Clustering analysis

We carried out several clustering and phylogenetic analyses to investigate the relative evolutionary affinities of the 83 strains included in the study. A maximum-likelihood phylogeny was constructed based on SNPs. The SNPhylo pipeline was used to filter and align SNPs, as a first step in phylogeny construction [37]. The minor allele frequency was set to 0.025 and the max missing data filter was set to 0.1. The linkage disequilibrium filter was set to 0.9. This left 37,893 SNPs. The maximum-likelihood phylogeny and bootstrapping analysis (1000 replicates) was performed in R using the filtered SNP set and the phangorn R package. Neighbor-joining phylogenies were constructed using the “nj” function in the ape R package [38]. K-means clustering, an algorithm to classify objects into a predefined number of groupings, and Discriminant Analysis of Principal Components (DAPC) were performed using the find.clusters and dapc functions, as implemented in the R package adegenet [39]. These clustering analyses were carried out on different SNP data sets: (1) the entire 162,210 SNP data set to identify the most likely number of different genetic clusters in which the 83 *Trypanozoon* strains could be grouped; (2) several sub-sets of the main dataset that included either a smaller number of either SNPs or strains (see below). A-score optimization was performed to avoid over-fitting for each DAPC analysis, following the recommendations of the package authors [39].

### SNP sub-set classification

To identify subsets of SNPs that are especially useful and informative to classify an unknown strain to one of the identified genetic groups (see above), we carried out logistic regression and Sparse Linear Discriminant Analysis (SLDA). Starting with the main set of SNPs and the clusters identified by k-means clustering, we used a four-fold cross validation strategy, where four replicates of the data were used, with each having one-fourth of the initial data removed from the analysis to avoid over-fitting. This resulted in four data sets, each comprised of three quarters of the total data. Within each sub-dataset, dichotomized variables were generated to indicate the cluster label for each individual. An LD filtering step was carried out using the PLINK clump procedure (LD threshold = 0.2), to eliminate highly similar SNPs.

On each data set, we carried out logistic regression analysis to identify SNPs significantly associated with each of the genetic clusters identified by k-means clustering (p-value < 0.05). This was done using the software PLINK association procedure [40]. SNPs passing the significance and LD thresholds from all binary models were then aggregated to construct a discriminant function to classify strains using SLDA, as implemented in R (Package sparseLDA). The SLDA was repeated 1000 times, in each of the four sub-dataset, and selecting the top 10 SNPs with the greatest contribution for all the discriminant functions. SNPs were ranked based on their frequency of selection by SLDA across replicates, which reflected their utility in classifying strains into one of the original clusters.

We then examined the number of unique genotypes obtained only by considering combinations of the top-ranked SLDA-SNPs. Starting with the top ten ranked SLDA-SNPs, we concatenated the genotypes for each strain and calculated the number of unique combination genotypes obtained, using R. This procedure was repeated while adding the next highest-ranking SNPs until 83 unique combination genotypes were obtained.

To further test the classifying ability of the SLDA-selected SNPs (SLDA-SNPS), we repeated the DAPC and k-means clustering analysis on the top X ranked SLDA-SNPs, where X is equal to 10,000, 1000, 500, 250, 100, 50, 20, or 10 out of the total 31,164 SLDA-SNPs. We compared the clustering results obtained when using these eight subsets of SLDA-SNPs to the entire 162,210 SNP dataset by calculating the percentage of overlap in cluster membership for each strain, then calculating the average percentage for all strains. As an example, consider if Strain-X is assigned to cluster 1 based on the whole dataset, and cluster 3 based on the top 10 SLDA-SNPs (the clusters are arbitrarily named). The number of other strains assigned to both cluster 3 (based on the top 10 SLDA-SNPs), and cluster 1 (based on the entire SNP dataset), is 4. The total number of strains assigned to cluster 3 is 5 (not counting Strain-X). The percentage of overlap is 80% (4/5) for Strain-X. This metric is calculated for all strains, measuring how frequently the strains are grouped together in different k-means analyses.

To further assess the classification ability of the SLDA-SNPS we carried out an additional test. First, we removed 21 randomly selected strains from the original dataset. Then, we carried out DAPC analyses with each of the eight SLDA-SNP subsets, using the cluster assignment from the k-means clustering analysis done on the whole data set (giving data tables consisting of, for example, 62 strains by the top 10 SLDA-SNPs, 62 strains by the top 20 SLDA-SNPs, etc.). Then, we used the predict.dapc function of the adegenet R package to assign the 21 previously removed strains to a cluster, based on the discriminant function produced by the new DAPC. The assignment was considered successful if the correct cluster was given the highest probability score by the predict.dapc function. This process was repeated with 10 sets of 21 randomly selected strains for each of the 8 SLDA-SNP subsets.

## Results

We jointly analyzed the genome sequences of 83 trypanosome strains, 25 of which were previously unpublished. To characterize genomic variation among these strains, we identified 931,876 SNPs. Filtering for minor allele frequency (0.05), missing data, indels and informativeness yielded 162,210 SNPs for clustering and phylogenetic analysis. This reduced set is smaller than the one identified by Sistrom et al. 2014 [10] (608,501 SNPs). This is likely due to our avoidance of mapping to variant surface glycoprotein (VSG) genes and other repetitive sequences (see methods). In addition, we only mapped to sequences on the major chromosomes of *Tbb*, and applied a minor allele frequency filter.

### Phylogenetic and cluster analysis

**Fig 1:**
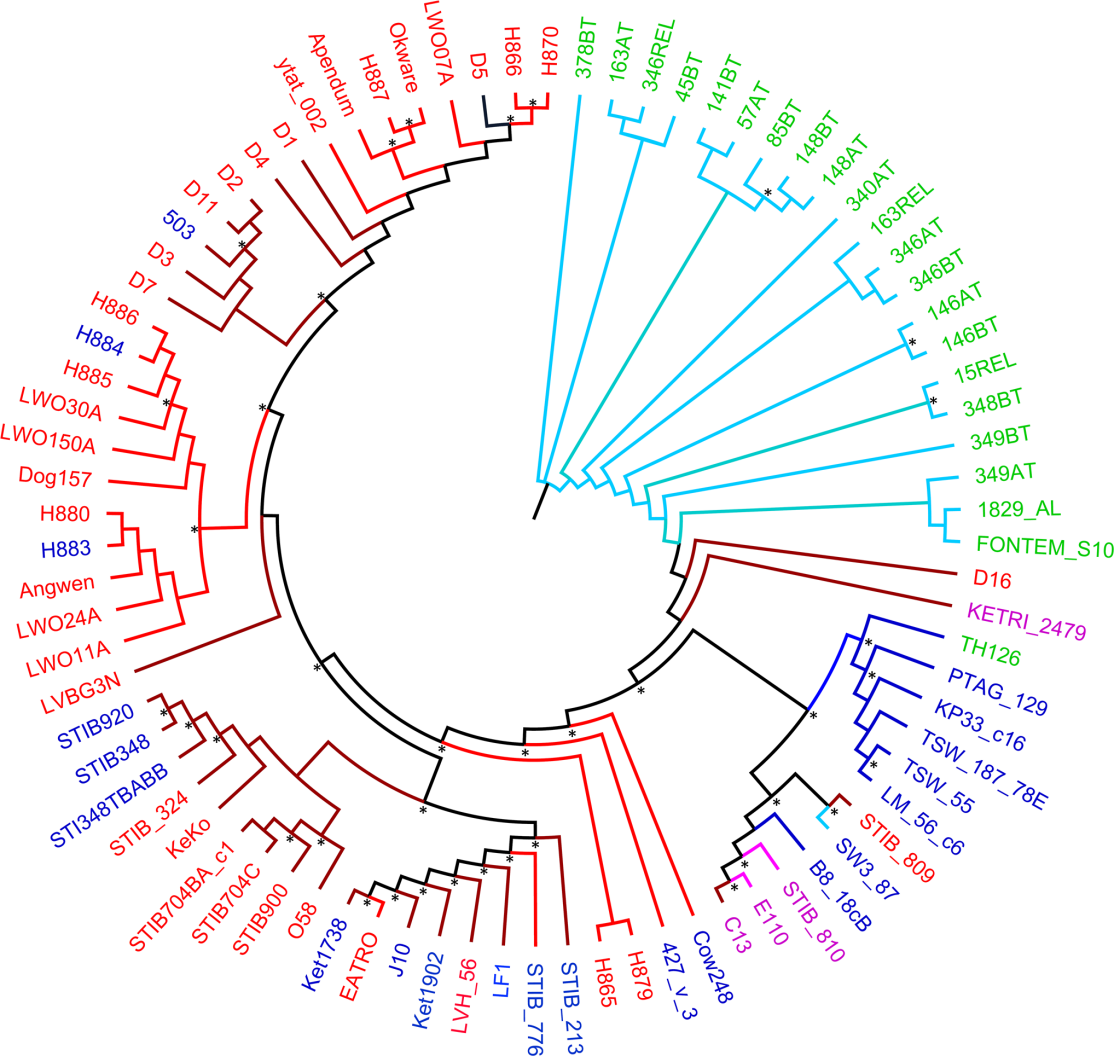
Maximum-likelihood phylogeny of 83 *Trypanosoma brucei* strains. Based on 37,893 concatenated SNPs obtained by using SNPhylo and Phangorn in R. Color of strain names indicates their named sub-species: blue: *Tbb*, red: *Tbr*, green: *Tbg-1* and *Tbg-2*, purple: *Tev*. Color of the branches indicate the geographic origin of each strain: dark blue: Western Africa, light blue: Democratic Republic of the Congo, light red: Uganda, dark red: Eastern Africa, Purple: outside of Africa. See S1 Table for geographic locations. Internal branches are black if they connect two branches of different colors. An asterisk identifies nodes with bootstrap support above 80%.

We used multiple phylogenetic and clustering methods to clarify the underlying genetic relationship of the 83 African trypanosome strains. We constructed a phylogenetic tree by first thinning the SNPs with more than 90% correlation to account for linkage, leaving 37,893 SNPs. The SNPhylo pipeline was used to concatenate and align the SNPs, and the phangorn R package was used to perform maximum-likelihood and bootstrapping analyses (Fig 1). Bootstrap percentages are shown on the phylogeny nodes. The strains in our study were classified as originating from Western Sub-Saharan Africa (Ivory Coast, Cameron, and Nigeria), the Democratic Republic of the Congo (DRC), Uganda, Eastern Sub-Saharan Africa (any point east of Uganda, within the endemic region of *Tbr*), or outside of Africa (*Tev* strains Type A: E110 and STIB 810). The Western strains are confined to two of the major clades, indicating some geographic separation among strains. In contrast, the *Tbb* and *Tbr* strains are broadly paraphyleitc. All *Tbg-1* strains are in a single clade, while the *Tbg-2* (Th126) is the sister taxon of a clade including other *Tbb* strains from Western Africa. This is likely a reflection of shared ancestral polymorphisms between some *Tbb* strains and this *Tbg-2* strain, confirming previous studies [8,10]. For *Tev*, the 3 type A strains cluster in a clade with *Tbb* strains, while the single type B strain in this study (KETRI 2479) falls in a relatively distant branch of the ML tree [7,41,42]. The results of the ML analysis are generally confirmed by the result of a neighbor-joining phylogeny based on the whole SNP dataset (162,210 SNPs, S1 Fig).

**Fig 2:**
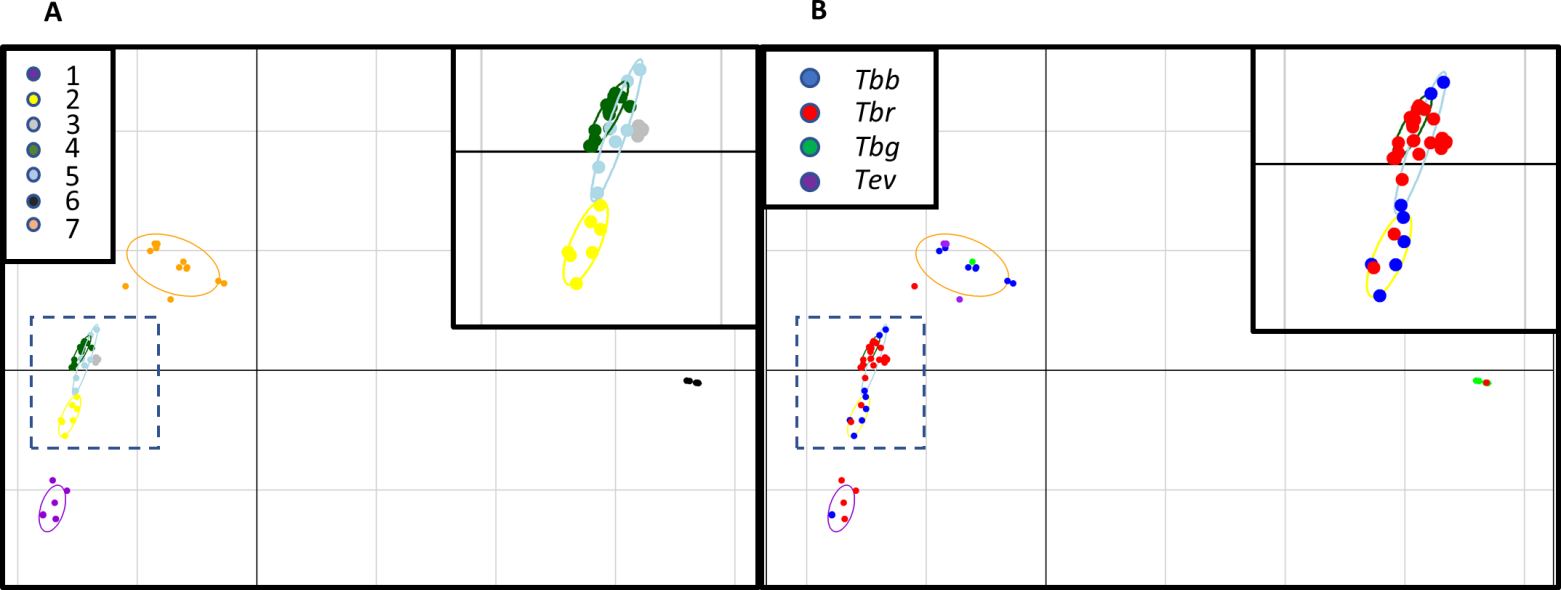
DAPC using the entire SNP data set. DAPC using the whole 162,210 SNP data set, with k = 7 clusters, based on k-means clustering. Scatter plot of the first two discriminant functions. Part A shows strains colored by cluster, and are connected by lines to the cluster’s centroid. The region enclosed by the dashed square is expanded in the inset for clarity. Part B shows the same data as in A, but with the strains colored according to their named taxon. The circles representing the clusters are the same as in part A for comparison.

To complement these analyses, we performed multivariate analyses on the whole SNP dataset (N = 162,210). We used Discriminant Analysis of Principal Components (DAPC), as it provides a way to visualize differences between clusters of strains. K-means clustering based on principal components built from SNPs was used to group the strains (indicated in S1 Table). This analysis indicated 7 to 11 distinct genetic clusters were present based on Bayesian Information Criterion (BIC) metrics (S2 Fig). Since BIC values were similar for K = 7–11, we present the results for the DAPC for k = 7 in Fig 2. DAPC calculates discriminant functions that optimally distinguish the clusters. These clusters are generally well separated, suggesting the k-means groupings reflect the underlying genetic structure of the samples. The first axis (x-axis) distinguishes cluster 6 (see S1 Table for cluster assignments) from the rest of the clusters. This cluster mainly contains the *Tbg-1* strains (see Fig 2B). Strain D16, a *Tbr* isolate, is also placed in this cluster. While its placement in mainly *Tbg* cluster is unusual, we have no reason to think our sample is mislabeled or contaminated, as D16 is SRA positive. The most likely explanation is that this *Tbr* strain is genetically close in terms of genome wide polymorphisms to the ancestral *Tbb* strain that also gave rise to the *Tbg* strains. The second axis distinguishes the remaining clusters from each other. While clusters 3, 4, and 5 are in close proximity, the BIC indicates the data is best explained by 7 instead of 6 or fewer clusters, arguing against collapsing the data into fewer clusters. None of the groups contain samples exclusively from a single named sub-species or a geographic location. However, all 4 *Tev* strains are found in cluster 7, in contrast with the results of the phylogenetic analyses, where the type A strains cluster together while the Type B strain is included in a different cluster. Every cluster except for cluster 6 (the mainly *Tbg-1* cluster) contains at least one *Tbr* and *Tbb* strain. Thus, while DAPC detected genetic structure in the 83 strains, as it identifies at least 7 distinct genetic clusters, this structure does not coincide with the traditional taxonomy, with the exception of *Tbg-1*. The DAPC for K = 8 through K = 11 are qualitatively similar (see S3 Fig).

### Diagnostic SNP discovery

We asked if a smaller number of SNPs could be useful in measuring genetic diversity or diagnosing strain origin among the 83 strains analyzed. This can be useful for studies wishing to carry out genome level analyses on a large number of strains, or in the development of diagnostic tools. To assess the amount of information that could be extracted from the fewest number of SNPs and still reflect the information gathered when using tens of thousands of SNPs, we took the approach of ranking SNPs based on their ability to differentiate strains, using Sparse Linear Discriminant Analysis (SLDA, see Methods for complete details). SLDA selected 31,164 SNPs at least once. The top 1500 most frequently picked SNPs were selected in more than 50% of the times, suggesting that a small number of SNPs (less than 4% of the initial 31,164 SNPs) makes a disproportionately large contribution to classifying strains into the 7 pre-defined clusters.

**Fig 3:**
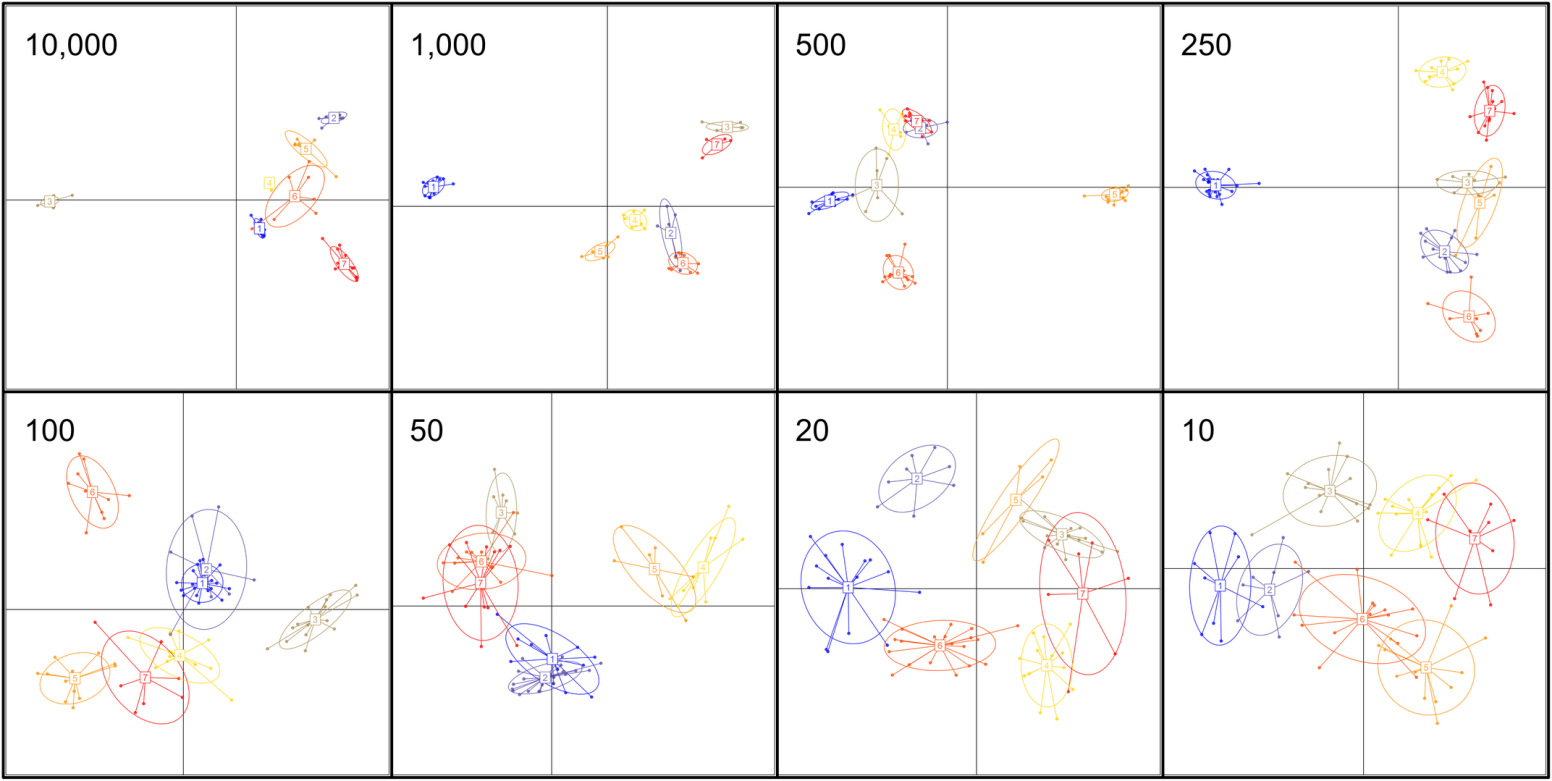
DAPC results for the eight SLDA-SNPs sets. Number of SLDA-SNPs indicated on each sub-figure. Scatter plots use the first two discriminant functions. Strains from the same cluster have the same color, and are connected by lines to the cluster’s centroid. The cluster number is arbitrarily assigned for each analysis.

We then tested how well smaller subsets of SNPs performed based on their ranking in the SLDA study by using several metrics. Next, we performed DAPC and k-means clustering for 8 different data sets, using the top-ranked 10,000, 1000, 500, 250, 100, 50, 20, or 10 SNPs (SLDA-SNPs). Fig 3 shows the scatter plots of the first two discriminant functions for selected eight SLDA-SNP data sets. The results show that, when using datasets with fewer SNPs (as low as 10,000, Fig 3, upper left corner, for example), the clusters still tend to be separated, matching the pattern seen with the whole dataset (Fig 2), but with fewer SNPS the separation is reduced, and the clusters are less discrete (compare Fig 2 to Fig 3, for example). However, all DAPC analyses, regardless of SNP numbers, differentiate strains into seven groups. Interestingly, even using only the top 10 SLDA-SNPs result in well distinguished clusters (Fig 3, bottom right corner).

**Fig 4:**
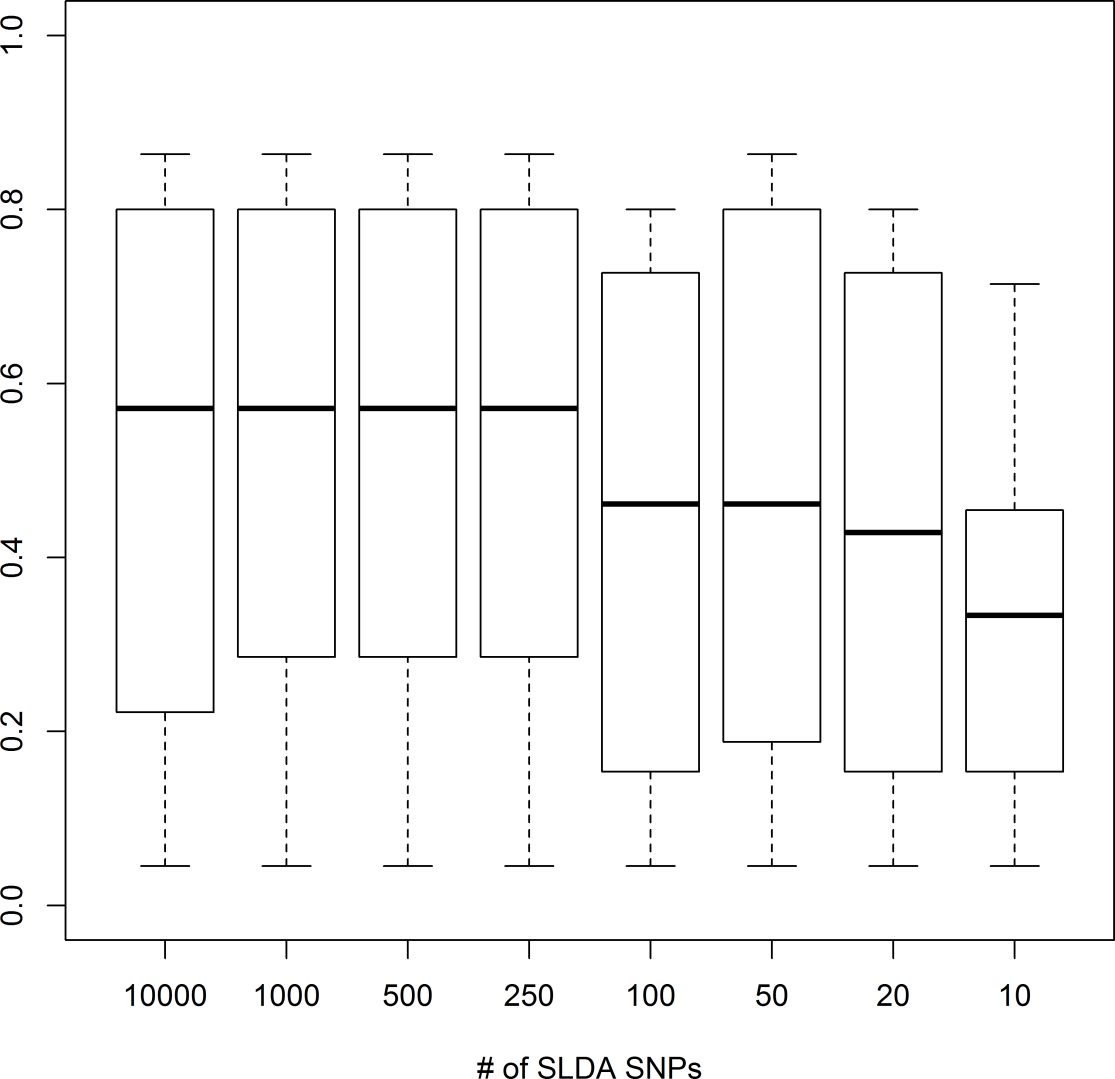
The median co-occurence of strains in clusters generated by DAPC. Clusters generated using the whole SNP dataset and DAPC using the number of SLDA-SNPs indicated on the x-axis. Boxes indicate the upper and lower quartiles, and whisker length is equal to 1.5 times the interquartile range.

To further assess the congruence of the DAPC results on the SLDA-SNPs data sets with the one from the whole data set, we checked how many times a certain strain grouped together with the same strains in the SLDA-SNPs and in the full SNP dataset DAPC analyses. We did this by examining each strain in the a SLDA-SNP data set, and calculating the percentage of strains that co-occur with that strain in its cluster assigned by the SLDA-SNP DAPC and the full data-set DAPC (See Methods for details). Fig 4 shows the percentage of strains co-occurring in the same cluster averaged for each SLDA-SNP. This metric gives an indication of how frequently strains are grouped together in the same cluster, using the smaller versus the larger SNP dataset. The percentage is 57% while using 10,000 to 250 SLDA-SNPs, and 33%, when using as few as 10 SLDA-SNPs.

**Fig 5:**
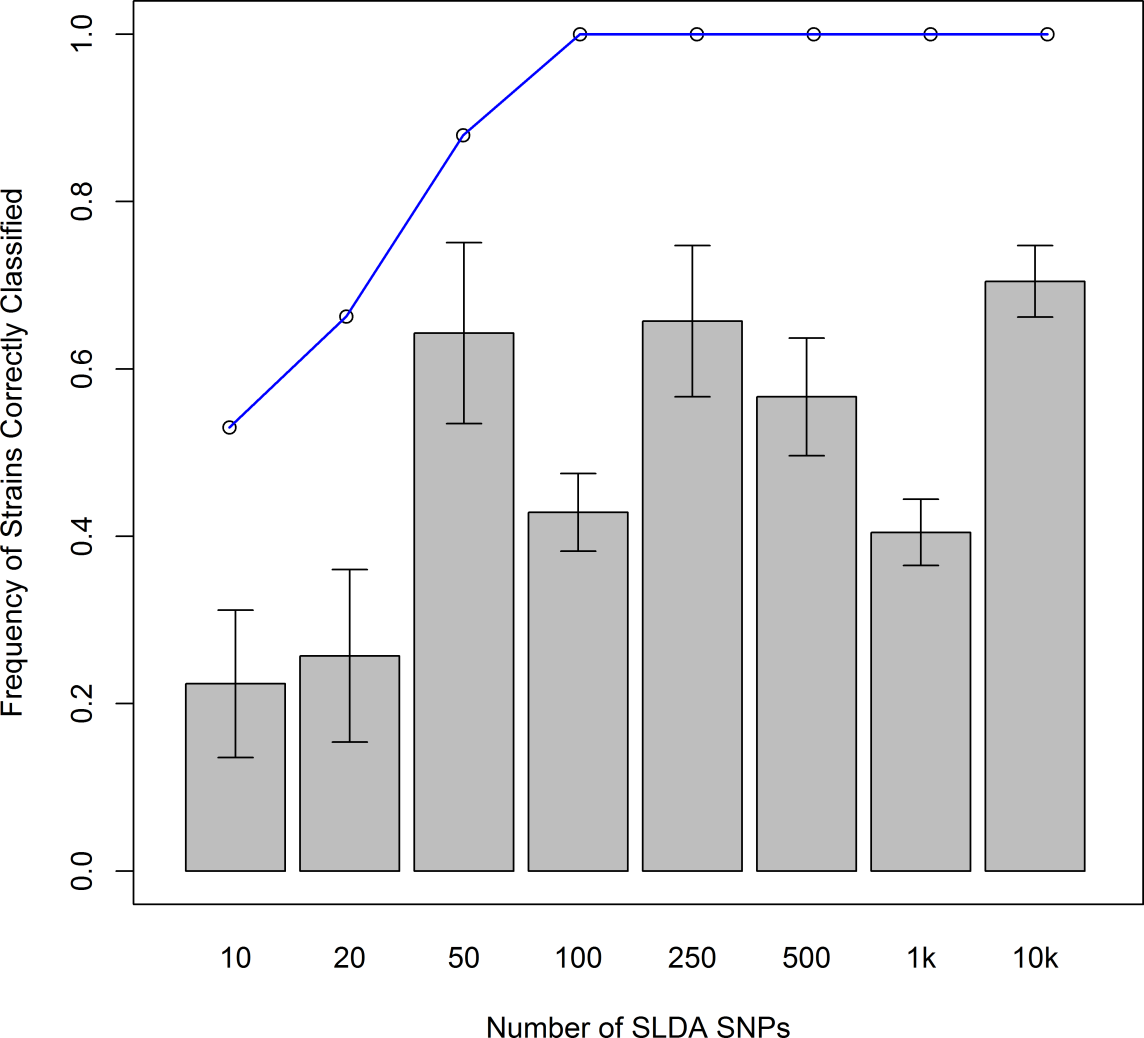
Average number of correctly classified strains. Average number of strains (+/- standard error of the mean) correctly classified using the DAPC results from the eight different SLDA-SNPs datasets (number of SNPs used indicated on the x-axis). Averages are based on 10 runs, and the highest score possible per run is 21. The blue line indicates the number of unique genotypes present when using the number of SLDA-SNPs on the x-axis, expressed as a percentage (83 unique genotypes are possible). See methods for further details.

As a test of the classification ability of the SLDA-SNPs, we repeated the DAPC analysis using each of the 8 SLDA-SNP sets after first removing 21 strains (approximately 25% of the strains) to see how often the removed strains fell into one of the 7 clusters they were assigned to by the full data set. We used the “predict.dapc” function to use the discriminant analysis made by DAPC to classify the excluded strains. This process was repeated with 10 sets of 21 randomly chosen strains. The average number classified correctly for each SLDA-SNP set is shown in Fig 5. The results vary for different numbers of SNPs, but at least 40% of the strains are classified correctly when using at least 50 SNPs. This variability is likely due to randomness in the strains chosen for each test. While the SNPs are ranked based on their ability to distinguish the strains in our study, some SNPs may be better or worse at distinguishing certain strains that make up the test set.

We next examined the genotypes of each strain for the top-ranked SLDA-SNPs to see how many SLDA-SNPs were required to generate a unique combination of genotypes for each strain. The genotypes of the top 84 SLDA-SNPs combined to generate a unique genotype for each strain, suggesting that using SNPs ranked by SLDA provides a powerful way to identify strains using a select few informative SNPs (84 out of 162,210, less than 0.1%) that represents the genetic relationships of the strains based on whole genome data (Fig 5).

## Discussion

### Evolutionary relationships

Given the medical and economic importance of trypanosomiasis, we searched the genomic data for markers, which could be used to understand the evolutionary relationships among African trypanosomes. We identified 162,210 SNPs among the 83 *Trypanozoon* samples in our study. The phylogenetic and clustering analyses elucidate the evolutionary relationship among the strains (Fig 1) and group them in clusters of genetically similar strains (Fig 2), yet none of them reflect the traditional taxonomy implied by their taxonomic rank.

In the ML phylogeny (Fig 1) none of the sub-species and species form monophyletic clusters, according to their taxonomic rank. The only exception is represented by the clade that grouped all *Tbg-1* strains, confirming their close genetic relationship, most likely due to their mostly asexual nature [15]. This reinforces the idea, suggested by others, that the different species or sub-species are actually morphotypes within the same genetic meta-population rather than distinct evolutionary lineages as is implied by their current taxonomic ranks. This is especially apparent for the *Tbr* strains, where they are frequently found in clades near *Tbb* strains, and more distant from other *Tbr* strains. For *Tev*, our conclusions are weakened by the fact that only 4 strains are included in this analysis. Interestingly, the three type A strains we analyzed (S1 Table) cluster together, with a *Tbb* strain, as the next most closely related strain to that clade, while the only Type B strain (KETRI 2479) is in a relatively distant branch of the phylogeny.

Geographic origin is reflected weakly in the ML phylogeny. The strains from Western Africa (shaded in light or dark blue in Fig 1) are found in two of the major branches, while the Eastern African strains are found throughout the phylogeny. One of these branches contains the *Tbg-1* strains, which come largely from the DRC. In this instance, sub-species classification as *Tbg-1* rather than geographic origin may drive membership in this clade. The only other strain from DRC (SW3_87, a *Tbb* strain) is found in the clade with the other Western African strains, supporting this observation. *Tbb* and *Tbr* strains from Eastern Africa are found throughout the rest of the phylogeny and do not seem to group according to country of origin. Therefore, while there does appear to be a geographic signal in the data, it does not fully explain similarities between the strains, and none of the regions could be assigned exclusively to a single clade. Sistrom et al. [10], who performed a comparative genomic analysis of 23 *Tbr* and *Tbb* strains, including some of those studied in our analysis, also found some evidence of geographic structure. They performed a clustering analysis and found a significant association between geographic origin and cluster membership. As an example, all of the strains from the Ivory Coast in our analysis were also included in [10]. In both studies, all of the Ivory Coast strains were assigned to one cluster. As another example, our analysis contains 8 strains of Tanzanian origin (4 of which were included in [10]) and all but one are included in same DAPC group. The strains from Uganda and DRC, which make up the majority of the strains in our study, though, do not form monophyletic clades. However, strains from Western sub-Saharan Africa are generally well separated from the Eastern strains. Taken together, geographic origin appears to contribute to the genetic structure of the African trypanosomes analyzed, but does not completely explain it.

The phylogenetic analyses were complemented by the results of the multivariate analyses, which focus on identifying how many genetically distinct groups of strains are present in our dataset, regardless of their phylogenetic relationships. The DAPC results group strains in 7 clusters, indicating the presence of genetic differentiation among them. However, as in the phylogenetic analyses, the strains do not group according to their traditional taxonomic classification or geographic origin. Clusters tend to include more than a single species or sub-species, although cluster 6 contains all *Tbg-1* strains and includes only one other non-*Tbg* strain (strain D16). Cluster 7 groups all four *Tev* strains analyzed in this study, a result at odds with the topological assignment in Fig 1, where the only Type B *Tev* strain falls in a differ clade from the other 3 *Tev* strains (S1 Table). However, cluster 7 also contains *Tbb* and *Tbr* strains, ranging from the Ivory Coast to Ethiopia, so the members of this cluster are not very similar due to geography or taxonomy. A larger sample size of *Tev* strains will be able to more fully address the question of *Tev* phylogeny among the different types. A recent genome analysis based on a few *Tev* strains confirmed the origin of type B and A from different *Tbb* strains. This result has been recently corroborated by a microsatellite survey of 41 *Tve* isolates from Kenya [43]. In general, a revision of the taxonomic ranks of the African trypanosomes that reflects both their evolutionary history and their adaptation [6] is warranted.

### Marker selection

While neither geography nor the traditional sub-species/species designations fully explain the patterns of genetic structure observed in the African trypanosomes strains in this study, the observation of genetic structure can be used to develop assays based on strain-specific markers. These assays can be used, for instance, to track the origins of new outbreaks by genotyping a large number of strains with relatively little effort. This type of tool can also facilitate population level analyses by enabling researchers to look at the pattern and amount of spread over space and time for a large number of strains, allowing for quick identification of possible recombinant genotypes between different pathogenic strains.

However, classifying and tracking strains cannot rely on whole genome comparisons, as whole-genome sequencing of parasite populations is still expensive and non-trivial. Our analysis suggests that careful selection of SNPs, using statistical methods such SLDA, can yield comparable information to genome-wide data. Tests of the ability of SNPs to assign strains to predefined clusters showed that using 10,000 SLDA-SNPs resulted in an average of 13 out of 21 correct classifications per test, though this number dropped to approximately 5 per test when using 10 to 20 SLDA-SNPs (Fig 5). Although apparently disappointing, the results of this type of analyses are encouraging, when considering that this test is quite conservative, using only the information from two-thirds of the strains, and the remaining third for testing. Additional strains would likely increase the classification power. The informativeness of the SLDA-SNPs is further validated by the observation that 84 SLDA-SNPs are sufficient to distinguish each of the strains in our study, when using them in genotypic combinations rather than as isolated SNPs. Using as few as 50 SLDA-SNPs still yielded 73 unique genotypes (Fig 5). These results are encouraging since we could distinguish very closely related strains and coming from the same location, such as the *Tbg* strains analyzed in this study (see [32]). This finding could lead to the development of field friendly barcode-based assay, as has been done for other parasites, such as *Plasmodium falciparum* [44], where TaqMan genotyping assays have been developed using only 24 SNPs [44,45].

In conclusion, this analysis takes advantage of genomic data coupled with statistical methods not generally used for strain identification to select a small subset of SNPs that have similar information content as the whole genome data to uniquely identify strains. This approach can be used in a variety of contexts and with different types of organisms, as long as genome data for a representative group of individuals is available, allowing the development of efficient and relatively inexpensive ways to screen for genomic variation a large number of samples, while still retaining the information content provided by whole genome analyses.

## Supporting information

S1 Table List of strains. Tbb = *Trypanosoma brucei brucei*, Tev-Type A = *Trypanosoma evansi* Type A, Tev-Type B = *Trypanosoma evansi* Type B, Tbg-1 = *Trypanosoma brucei gambiense* group 1, Tbg-2 = *Trypanosoma brucei gambiense* group 2, Tbr = *Trypanosoma brucei rhodesiense*. The final column lists the reference where the genome was first published. NA indicates this is the first time the genome is described.
(XLS)

S1 Fig Unrooted Neighbor-joining phylogeny of all 83 Tb strains. Based on 162,210 SNPs. Color of strain names indicates the sub-species: blue: *Tbb*, red: *Tbr*, green: *Tbg*, purple: *Tev*.
(TIFF)

S2 Fig Comparison of Bayesian Information Criterion (BIC) for varying number of clusters identified by k-means clustering of SNP-based principal components. BIC plotted for k = 1–40. Clustering based on the 162,210 SNP dataset.
(TIF)

S3 Fig DAPC using the whole 162,210 SNP data set, with k = 11 clusters, based on k-means clustering. Part A shows strains colored by cluster, and are connected by lines to the cluster’s centroid. The region enclosed by the dashed square is expanded in the inset for clarity. Part B shows the same data as in A, but with the strains colored according to their named taxon. The circles representing the clusters are the same as in part A for comparison.
(TIF)

## References

[1] Lai DH, Hashimi H, Lun ZR, Ayala FJ, Lukes J. Adaptations of *Trypanosoma brucei* to gradual loss of kinetoplast DNA: *Trypanosoma equiperdum* and *Trypanosoma evansi* are petite mutants of *T*. brucei. Proc Natl Acad Sci USA. 2008;105 (6):1999–2004. doi: 10.1073/pnas.0711799105 18245376 10.1073/pnas.0711799105 PMC2538871

[2] Birhanu H, Gebrehiwot T, Goddeeris BM, Buscher P, Van Reet N. New *Trypanosoma evansi* Type B isolates from Ethiopian dromedary camels. PLoS Negl Trop Dis. 2016; 10(4): e0004556 doi: 10.1371/journal.pntd.0004556 27035661 10.1371/journal.pntd.0004556 PMC4818106

[3] Berberof M, David P-M, Etienne P. A receptor-like flagellar pocket glycoprotein specific to *Trypanosoma brucei* gambiense. Mol Biochem Parasitol 2001;113 (1):127–138. 11254961 10.1016/s0166-6851(01)00208-0

[4] De Greef C, Hamers R. The serum resistance-associated (SRA) gene of *Trypanosoma brucei* rhodesiense encodes a variant surface glycoprotein-like protein. Mol Biochem Parasitol. 1994;68:277–84. 7739673 10.1016/0166-6851(94)90172-4

[5] Symula RE, Beadell JS, Sistrom M, Agbebakun K, Balmer O, Gibson W, Aksoy S, Caccone A. *Trypanosoma brucei* gambiense group 1 is distinguished by a unique amino acid substitution in the HpHb receptor implicated in human serum resistance. PLoS Negl Trop Dis. 2012;6(7):e1728 doi: 10.1371/journal.pntd.0001728 22802982 10.1371/journal.pntd.0001728 PMC3393672

[6] Gibson W. Resolution of the species problem in African trypanosomes. Int J Parasitol 2007;37 (8–9):829–38. doi: 10.1016/j.ijpara.2007.03.002 17451719 10.1016/j.ijpara.2007.03.002

[7] Carnes J, Anupama A, Balmer O, Jackson A, Lewis M, Brown R, Cestari I, et al Genome and phylogenetic analyses of *Trypanosoma evansi* reveal extensive similarity to *T*. *brucei* and multiple independent origins for dyskinetoplasty. PLoS Negl Trop Dis. 2015;9 (1):e3404 doi: 10.1371/journal.pntd.0003404 25568942 10.1371/journal.pntd.0003404 PMC4288722

[8] Balmer O, Beadell JS, Gibson W, Caccone A. PLoS Negl Trop Dis. 2011;2 8;5(2):e961 doi: 10.1371/journal.pntd.0000961 21347445 10.1371/journal.pntd.0000961 PMC3035665

[9] Gibson WC, Marshall T. F de C, Godfrey DG. Numerical analysis of enzyme polymorphism: a new approach to the epidemiology and taxonomy of trypanosomes of the subgenus Trypanozoon. Adv. Parasitol. 1980;18:175–246. 7001872 10.1016/s0065-308x(08)60400-5

[10] Sistrom M, Evans B, Bjornson R, Gibson W, Balmer O, Mäser P, Aksoy S, Caccone A. Comparative Genomics Reveals Multiple Genetic Backgrounds of Human Pathogenicity in the *Trypanosoma brucei* Complex. Genome Biol Evol. 2014;10 5;6(10):2811–9. doi: 10.1093/gbe/evu222 25287146 10.1093/gbe/evu222 PMC4224348

[11] Sistrom M, Evans B, Benoit J, Balmer O, Aksoy S, Caccone A. De Novo Genome Assembly Shows Genome Wide Similarity between *Trypanosoma brucei brucei* and *Trypanosoma brucei* rhodesiense. PLoS One. 2016 2 24;11(2):e0147660 doi: 10.1371/journal.pone.0147660 26910229 10.1371/journal.pone.0147660 PMC4766357

[12] Mehlitz D, Zillmann U, Scott CM, Godfrey DG (1982) Epidemiological studies on the animal reservoir of Gambiense sleeping sickness. III. Characterization of Trypanozoon stocks by isoenzymes and sensitivity to human serum. Tropenmedizin und Parasitologie 33: 113–118. 6287687

[13] Gibson WC (1986) Will the real *Trypanosoma b*. *gambiense* please stand up. Parasitology Today 2: 255 15462856 10.1016/0169-4758(86)90011-6

[14] Jackson AP, Sanders M, Berry A, McQuillan J, Aslett MA, Quail MA, Chukualim B, Capewell P, MacLeod A, Melville SE, Gibson W, Barry JD, Berriman M, Hertz-Fowler C. The genome sequence of Trypanosoma brucei gambiense, causative agent of chronic human african trypanosomiasis. PLoS Negl Trop Dis. 2010 4 13;4(4):e658 doi: 10.1371/journal.pntd.0000658 20404998 10.1371/journal.pntd.0000658 PMC2854126

[15] Weir W, Capewell P, Foth B, Clucas C, Pountain A, Steketee P, Veitch N, Koffi M, De Meeûs T, Kaboré J, Camara M, Cooper A, Tait A, Jamonneau V, Bucheton B, Berriman M, MacLeod A. Population genomics reveals the origin and asexual evolution of human infective trypanosomes. Elife. 2016 1 26;5:e11473 doi: 10.7554/eLife.11473 26809473 10.7554/eLife.11473 PMC4739771

[16] Lun ZR, Brun R GW. Kinetoplast DNA and molecular karyotypes of *Trypanosoma evansi* and *Trypanosoma equiperdum* from China. Mol Biochem Parasitol. 1992;50(2):189–96. 1311051 10.1016/0166-6851(92)90215-6

[17] Picozzi K, Fevre EM, Odiit M, Carrington M, Eisler MC, Maudlin I, Welburn SC. Sleeping sickness in Uganda: a thin line between two fatal diseases. Bmj 2005;331 (7527):1238–41. doi: 10.1136/bmj.331.7527.1238 16308383 10.1136/bmj.331.7527.1238 PMC1289320

[18] Richardson JB, Evans B, Pyana PP, Van Reet N, Sistrom M, Buscher P, et al Whole genome sequencing shows sleeping sickness relapse is due to parasite regrowth and not reinfection. Evol Appl 9 2016;(2):381–93. 10.1111/eva.12338 PMC4721075 26834831

[19] Echodu R, Sistrom M, Bateta R, Murilla G, Okedi L, Aksoy S, et al Genetic diversity and population structure of *Trypanosoma brucei* in Uganda: implications for the epidemiology of sleeping sickness and Nagana. PLoS Negl Trop Dis. 2015 2 19;9(2):e0003353 doi: 10.1371/journal.pntd.0003353 25695634 10.1371/journal.pntd.0003353 PMC4335064

[20] Fraga J, Fernandez-Calienes A, Montalvo AM, Maes I, Deborggraeve S, Buscher P, Dujardin JC, et al Phylogenetic analysis of the *Trypanosoma* genus based on the heat-shock protein 70 gene. Infect Genet Evol 2016;43:165–72. doi: 10.1016/j.meegid.2016.05.016 27180897 10.1016/j.meegid.2016.05.016

[21] Clemmensen L, Hastie T, Witten D, Ersboll B. Sparse Discriminant Analysis. Technometrics. 2011; 53(4):406–413.

[22] Wu MC, Zhang L, Wang Z, Christiani DC, Lin X. Sparse linear discriminant analysis for simultaneous testing for the significance of a gene set/pathway and gene selection. Bioinformatics. 2009 5 1;25(9):1145–51 doi: 10.1093/bioinformatics/btp019 19168911 10.1093/bioinformatics/btp019 PMC2732305

[23] Zhang M, Lin Y, Wang L, Pungpapong V, Fleet JC, Zhang D. Case-control genome-wide association study of rheumatoid arthritis from Genetic Analysis Workshop 16 using penalized orthogonal-components regression-linear discriminant analysis. BMC Proc. 2009 12 15;3 Suppl 7:S17 20018006 10.1186/1753-6561-3-s7-s17 PMC2795913

[24] Kang M, Kim DC, Liu C, Gao J. Multiblock discriminant analysis for integrative genomic study. Biomed Res Int. 2015;2015:783592 doi: 10.1155/2015/783592 26075260 10.1155/2015/783592 PMC4450020

[25] Schwender H, Ickstadt K, Rahnenführer J. Classification with high-dimensional genetic data: assigning patients and genetic features to known classes. Biom J. 2008 12;50(6):911–26 doi: 10.1002/bimj.200810475 19067340 10.1002/bimj.200810475

[26] Pennisi E. Pocket DNA sequencers make real-time diagnostics a reality. Science. 2016; 2 19;351(6275):800–1 doi: 10.1126/science.351.6275.800 26912872 10.1126/science.351.6275.800

[27] Njiru ZK, Constantine CC, Guya S, Crowther J, Kiragu JM, Thompson RC, Dávila AM. The use of ITS1 rDNA PCR in detecting pathogenic African trypanosomes. Parasitol Res. 2005 2;95(3):186–92. Epub 2004 Dec 24. PubMed doi: 10.1007/s00436-004-1267-5 . 15619129 10.1007/s00436-004-1267-5

[28] Radwanska M, Claes F, Magez S, Magnus E, Perez-Morga D, Pays E, Büscher P. Novel primer sequences for polymerase chain reaction-based detection of Trypanosoma brucei gambiense. Am J Trop Med Hyg. 2002 9;67(3):289–95. 12408669 10.4269/ajtmh.2002.67.289

[29] Andrews, S. FastQC A Quality Control tool for High Throughput Sequence Data Available from http://www.bioinformatics.babraham.ac.uk/projects/fastqc/.

[30] Berriman M, Ghedin E, Hertz-Fowler C, Blandin G, Renauld H, Bartholomeu DC, et al The genome of the African trypanosome *Trypanosoma brucei*. Science. 2005 7 15;309(5733):416–22. PubMed doi: 10.1126/science.1112642 16020726 10.1126/science.1112642

[31] Langmead B, Salzberg SL. Fast gapped-read alignment with Bowtie 2. Nat Methods. 2012;9 (4):357–9. doi: 10.1038/nmeth.1923 22388286 10.1038/nmeth.1923 PMC3322381

[32] Van der Auwera GA, Carneiro MO, Hartl C, Poplin R, Del Angel G, Levy-Moonshine A, et al From FastQ data to high confidence variant calls: the Genome Analysis Toolkit best practices pipeline. Curr Protoc Bioinformatics 2013;11 (1110):11 10 1–11 10 33. 10.1002/0471250953.bi1110s43 PMC4243306 25431634

[33] McKenna A, Hanna M, Banks E, Sivachenko A, Cibulskis K, Kernytsky A, et al The Genome Analysis Toolkit: a MapReduce framework for analyzing next-generation DNA sequencing data. Genome Res. 2010 9;20(9):1297–303. doi: 10.1101/gr.107524.110 20644199 10.1101/gr.107524.110 PMC2928508

[34] Danecek P, Auton A, Abecasis G, Albers CA, Banks E, DePristo MA, et al The variant call format and VCFtools. Bioinformatics 2011;27 (15):2156–8. doi: 10.1093/bioinformatics/btr330 21653522 10.1093/bioinformatics/btr330 PMC3137218

[35] Smit AFA, Hubley R, Green P. RepeatMasker Open-3.0 1996–2010 Available from http://www.repeatmasker.org.

[36] R Development Core Team. R: A language and environment for statistical computing. R Foundation for Statistical Computing, Vienna, Austria 2008 ISBN 3-900051-07-0, URL http://www.R-project.org.

[37] Lee TH, Guo H, Wang X, Kim C, Paterson AH. SNPhylo: a pipeline to construct phylogenetic trees from huge SNP data. BMC Genomics. 2014;15:162 doi: 10.1186/1471-2164-15-162 24571581 10.1186/1471-2164-15-162 PMC3945939

[38] Paradis E, Claude J, Strimmer K. APE: analyses of phylogenetics and evolution in R language. Bioinformatics. 2004;20: 289–290. 14734327 10.1093/bioinformatics/btg412

[39] Jombart T, Devillard S, Balloux F. Discriminant analysis of principal components: a new method for the analysis of genetically structured populations. BMC Genet 2010;11:94 doi: 10.1186/1471-2156-11-94 20950446 10.1186/1471-2156-11-94 PMC2973851

[40] Purcell S, Neale B, Todd-Brown K, Thomas L, Ferreira MA, Bender D, et al PLINK: a tool set for whole-genome association and population-based linkage analyses. Am J Hum Genet. 2007;81 (3):559–75. doi: 10.1086/519795 17701901 10.1086/519795 PMC1950838

[41] Borst P, Fase-Fowler F, Gibson WC. Kinetoplast DNA of *Trypanosoma evansi*. Mol Biochem Parasitol. 1987;2;23(1):31–8. PubMed . 3033499 10.1016/0166-6851(87)90184-8

[42] Claes F, Radwanska M, Urakawa T, Majiwa PA, Goddeeris B, Büscher P. Variable Surface Glycoprotein RoTat 1.2 PCR as a specific diagnostic tool for the detection of *Trypanosoma evansi* infections. Kinetoplastid Biol Dis. 2004;9 17;3(1):3 PubMed doi: 10.1186/1475-9292-3-3 15377385 10.1186/1475-9292-3-3 PMC521498

[43] Kamidid CM, Saarman NP, Dion K, Mireji PO, Ouma C, Murilla G, et al Multiple evolutionary origins of *Trypanosoma evansi* in Kenya. PLoS NTD, in press. 10.1371/journal.pntd.0005895 PMC5605091 28880965

[44] Daniels R, Volkman SK, Milner DA, Mahesh N, Neafsey DE, Park DJ, et al A general SNP-based molecular barcode for Plasmodium falciparum identification and tracking. Malar J 2008;7:223 doi: 10.1186/1475-2875-7-223 18959790 10.1186/1475-2875-7-223 PMC2584654

[45] Daniels R, Chang HH, Sene PD, Park DC, Neafsey DE, Schaffner SF, et al Genetic surveillance detects both clonal and epidemic transmission of malaria following enhanced intervention in Senegal. PLoS One 2013;8 (4):e60780 doi: 10.1371/journal.pone.0060780 23593309 10.1371/journal.pone.0060780 PMC3617153

