# Supplementary figures and images for: Genomic analyses of African Trypanozoon strains to assess evolutionary relationships and identify markers for strain identification
Source: PLoS Negl Trop Dis. 2017 Sep 29;11(9):e0005949. doi: 10.1371/journal.pntd.0005949 (PMC5636163; doi:10.1371/journal.pntd.0005949)

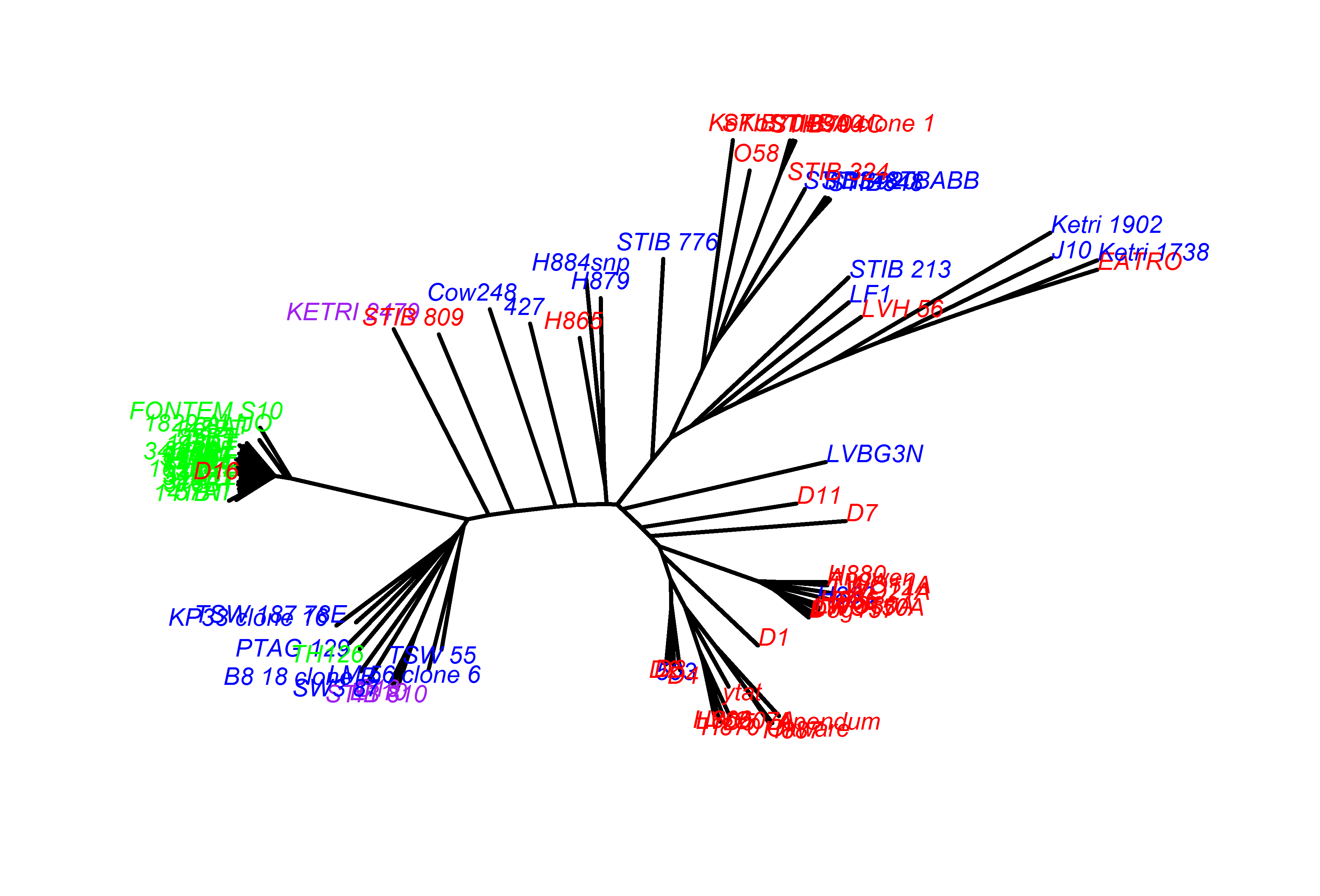

Supplement: S1 Fig — Based on 162,210 SNPs. Color of strain names indicates the sub-species: blue: Tbb, red: Tbr, green: Tbg, purple: Tev. (TIFF) [file pntd.0005949.s002.tiff]

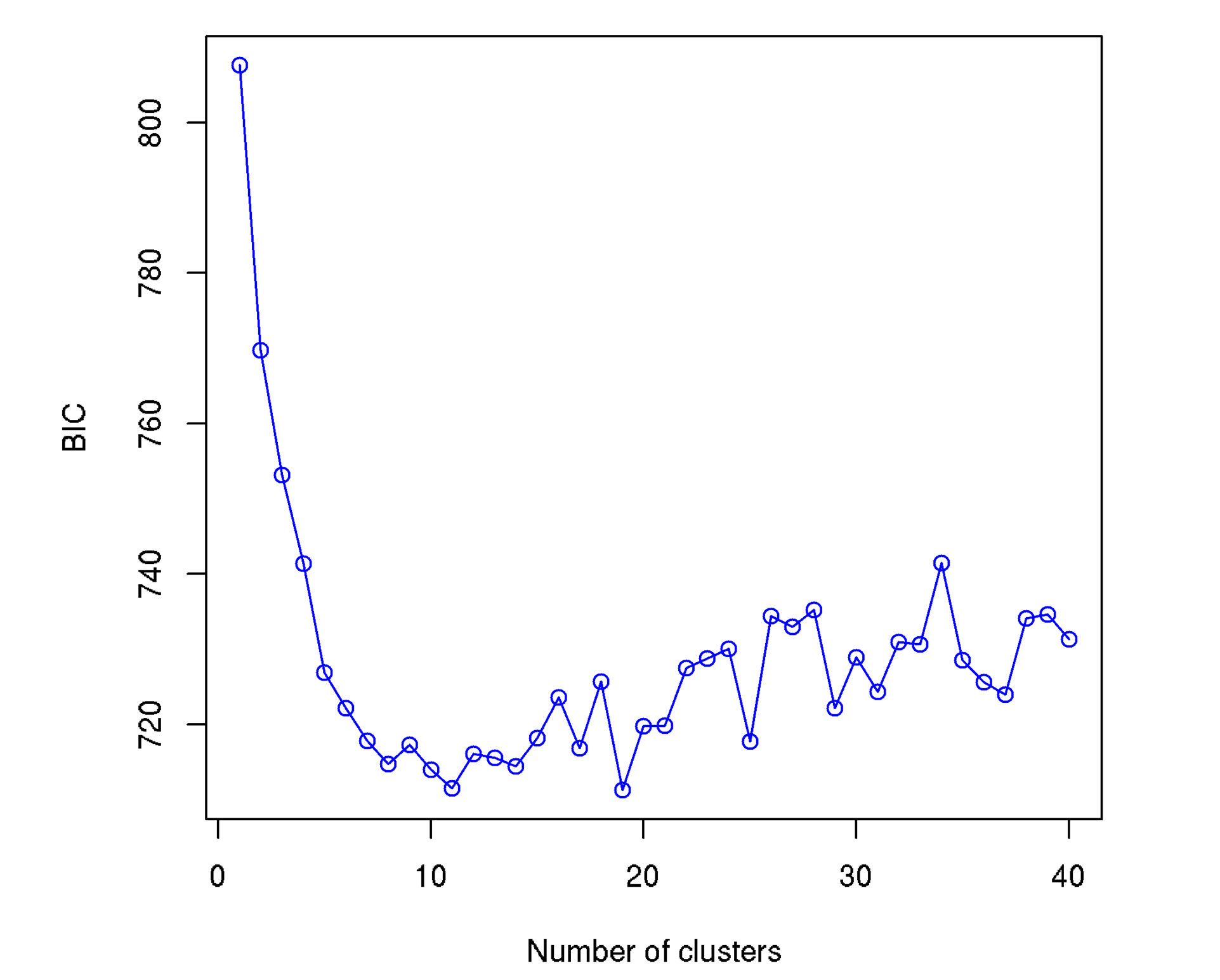

Supplement: S2 Fig — BIC plotted for k = 1–40. Clustering based on the 162,210 SNP dataset. (TIF) [file pntd.0005949.s003.tif]

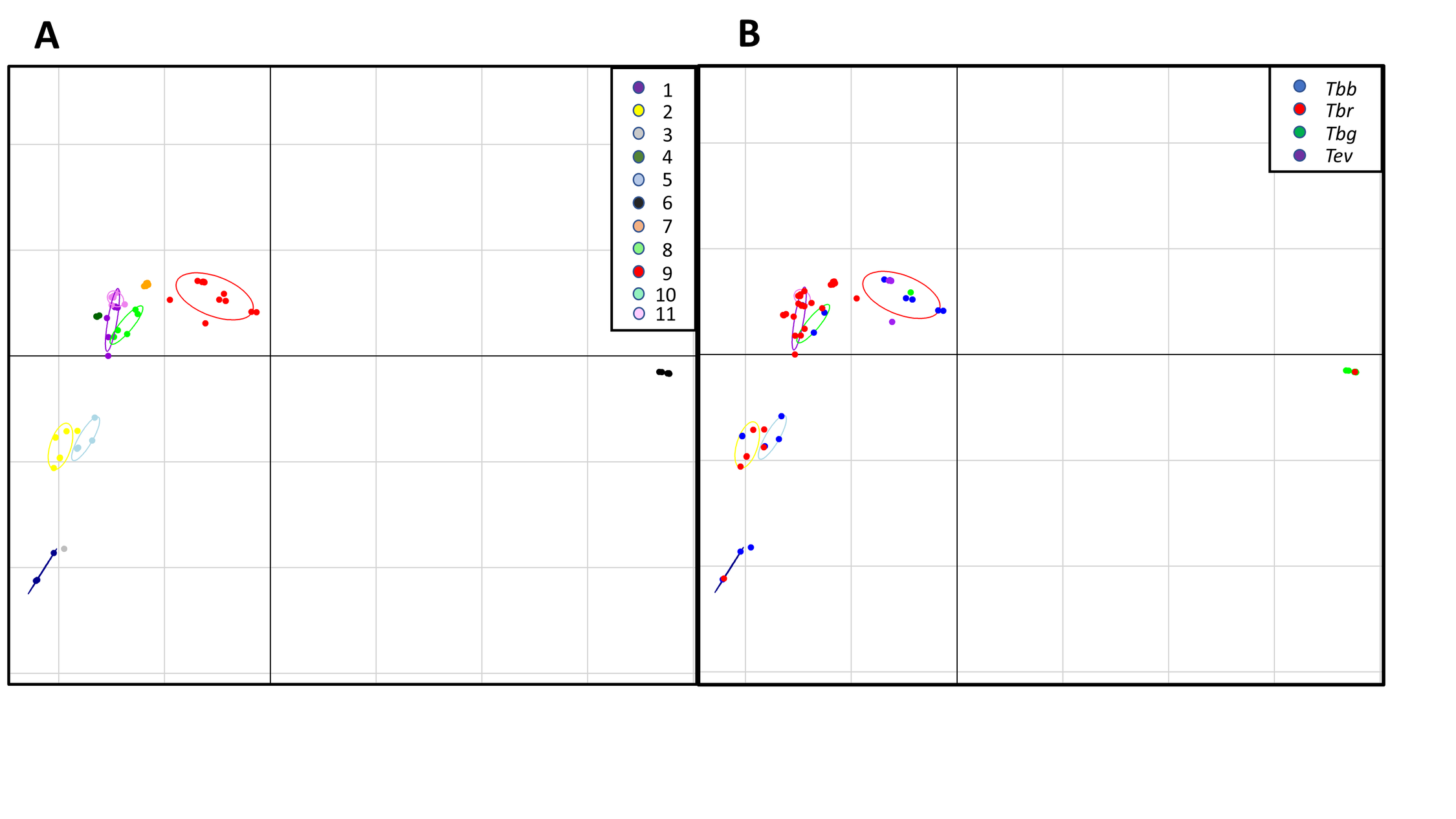

Supplement: S3 Fig — Part A shows strains colored by cluster, and are connected by lines to the cluster’s centroid. The region enclosed by the dashed square is expanded in the inset for clarity. Part B shows the same data as in A, but with the strains colored according to their named taxon. The circles representing the clusters are the same as in part A for comparison. (TIF) [file pntd.0005949.s004.tif]
